# A survey of synthetic nicotinamide cofactors in enzymatic processes

**DOI:** 10.1007/s00253-016-7500-1

**Published:** 2016-04-19

**Authors:** Caroline E. Paul, Frank Hollmann

**Affiliations:** Department of Biotechnology, Delft University of Technology, Julianalaan 136, 2628BL Delft, The Netherlands

**Keywords:** Biocatalysis, Biomimetics, Cofactor analogues, mNADHs, Synthetic nicotinamide cofactors, Oxidoreductases

## Abstract

Synthetic nicotinamide cofactors are analogues of the natural cofactors used by oxidoreductases as redox intermediates. Their ability to be fine-tuned makes these biomimetics an attractive alternative to the natural cofactors in terms of stability, reactivity, and cost. The following mini-review focuses on the current state of the art of those biomimetics in enzymatic processes.

## Introduction

Synthetic nicotinamide cofactors are biomimetics of natural cofactors required by oxidoreductases as redox equivalents. Although not new per se (Karrer and Stare 1937), these biomimetics have recently attracted increased attention in biocatalysis. The scope of this mini-review is to revisit the current state of the art of synthetic nicotinamide cofactors employed with oxidoreductases in various enzymatic processes. We will distinguish it from our previous review (Paul et al. 2014a) in which we described a historical view of the cofactors and applications in organic chemistry and medicine, and from our recent short succinct survey of our work (Hollmann and Paul 2015), by focusing on their application in enzymatic processes in the last two decades.

**Scheme 1:**
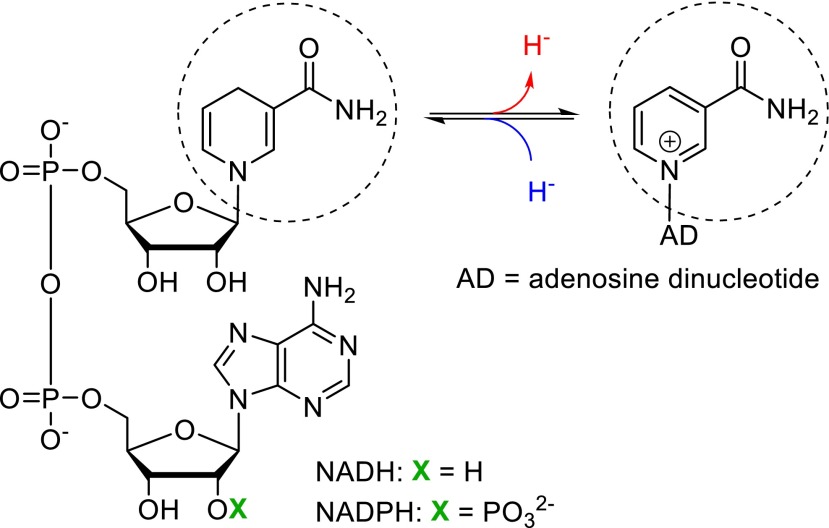
Structure of natural nicotinamide cofactors (reduced, *left*, and oxidized, *right*) giving or receiving two electrons in the form of a hydride at carbon C-4 of the pyridine ring

The key feature of the natural nicotinamide cofactor is its nicotinamide moiety that can act as an electron acceptor or donor through a hydride transfer (Scheme 1, dashed circle). The remaining structure of the molecule is the adenosine dinucleotide (AD) in its phosphorylated (NADP) or de-phosphorylated (NAD) form, which is important in two ways: first, the AD plays a major role for the cofactor recognition in the enzyme active site and correct positioning of the cofactor for optimal hydride transfer (Plapp 2010), second, oxidoreductases can be very selective toward the presence or absence of the phosphate group, thus playing a role in the regulation of the cellular metabolic pathways.

**Fig. 1:**
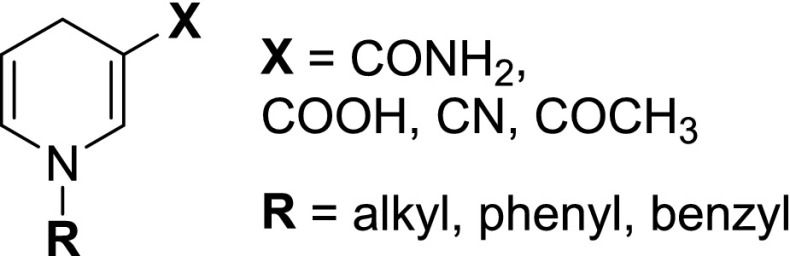
Structures of synthetic cofactor analogues

The study of nicotinamide cofactor analogues has been crucial for the elucidation of the mechanism of NAD(P)-dependent oxidoreductases (Anderson 1982; Mauzerall and Westheimer 1955) and has more recently progressed toward the actual use of analogues to improve enzymatic processes, which we will describe here in this mini-review. Synthetic nicotinamide cofactors are obtained purely from pyridine derivatives by chemical treatment. These analogues can be entirely redesigned to change their electrochemical properties through different pyridine substituents—X and R (Fig. 1). Over the past two decades, several analogues have been used in enzymatic processes, which will be described below.

## Biomimetics and dehydrogenases

**Fig. 2:**
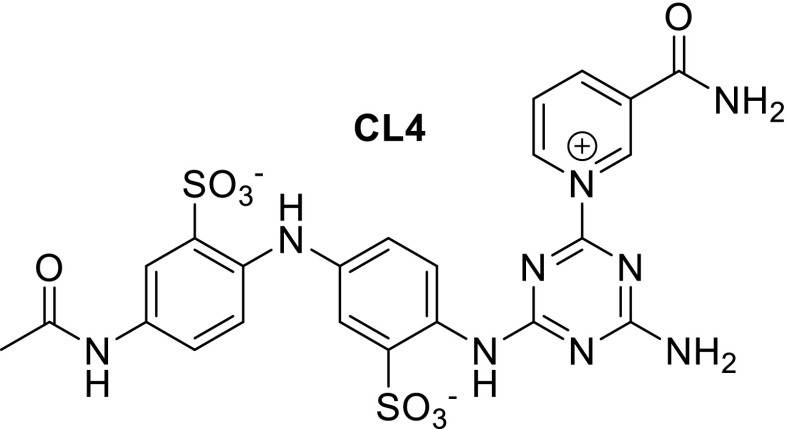
CL4 analogue used with HLADH

Various studies on dehydrogenases with substituent changes on the natural cofactors were performed before synthetic biomimetics were investigated. Ansell et al. developed analogues based on triazines and tested their activity with horse liver alcohol dehydrogenase (HLADH) (Ansell and Lowe 1999; Ansell et al. 1999a; Ansell et al. 1999b). The turnover numbers obtained remained very low with the synthetic CL4 analogue (Fig. 2).

**Scheme 2:**
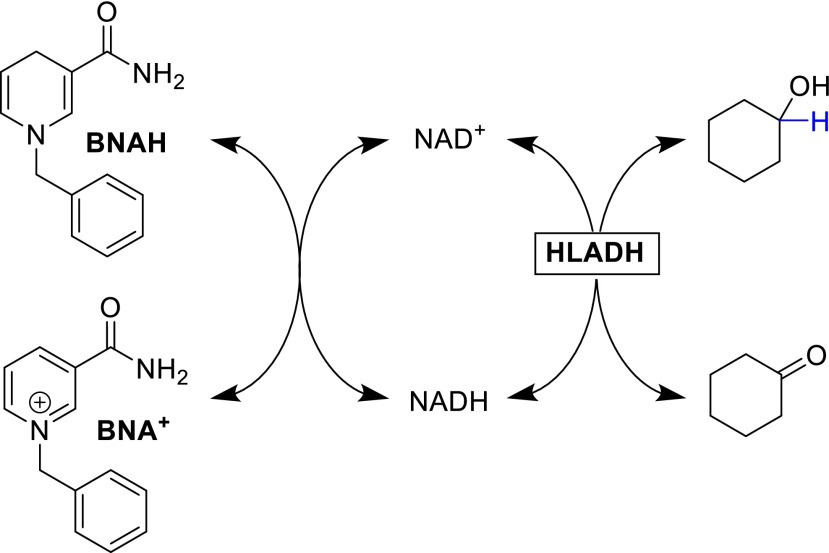
In situ regeneration of reduced and oxidized natural nicotinamide cofactors (in catalytic amounts) using the synthetic nicotinamide mimic BNAH (in stoichiometric amounts) to promote HLADH-catalyzed (stereospecific) reduction and oxidation reactions

A short while later, another report claimed ADHs accepting synthetic cofactors, which showed similar activity for 1-benzyl-1,4-dihydronicotinamide (BNAH) and nicotinamide mononucleotide (NMN) as with NADH (Lo and Fish 2002). However, upon closer inspection, these reports failed to clearly demonstrate the purity of the enzyme used in the enzymatic processes, as any trace amounts of natural cofactor can be recycled by the biomimetic, a process that was established and described by the group of Bryan Jones in 1976 (Taylor and Jones 1976). Furthermore, preliminary results from our own work using highly purified (nicotinamide cofactor-free) preparations of ADHs indicate that the majority of NAD(P)H-dependent ADHs does not accept the synthetic cofactors. Hence, the most likely explanation for the apparent enzyme activity is that in fact the in situ regeneration system proposed by Taylor and Jones occurs in these studies (Scheme 2).

## Biomimetics for flavin-dependent oxidoreductases

Next to the aforementioned dehydrogenases, which directly depend on the nicotinamide cofactors as redox partner for their reaction, flavin-dependent oxidoreductases often depend on NAD(P)H in a less direct way. Rather than directly participating in the reaction mechanism, the nicotinamide cofactor first reduces the (enzyme-bound) flavin prosthetic group (reductive half reaction). Then, in the oxidative half reaction, the reduced flavin either activates molecular oxygen (e.g. monooxygenases) or directly reduces the enzyme-bound substrate (e.g. old yellow enzymes and its analogues). Hence, the role of the nicotinamide cofactor is limited to reducing the flavin prosthetic group and, in principle, can be substituted by other reductants.

**Scheme 3:**
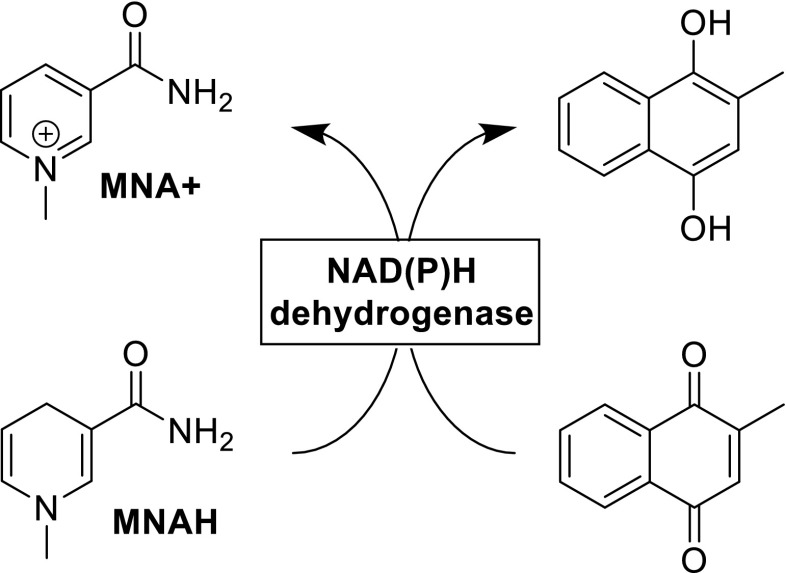
Reduction of menadione with MNAH using a NAD(P)H dehydrogenase

Friedlos et al. were the first to demonstrate that a simple short synthetic cofactor, methyl-1,4-dihydronicotinamide (MNAH), can perform as efficiently as NAD(P)H for the flavoprotein DT diaphorase (EC 1.6.99.2, 1.6.5.2), an NAD(P)H dehydrogenase (quinone), with a *k*_cat_ of approximately 6 × 10^−4^ min^−1^ and *K*_M_ of 200 μM for MNAH compared to 6.5 × 10^−4^ min^−1^ and 71 and 78 μM for NADH and NADPH, respectively (Friedlos et al. 1992). Thus, menadione could be reduced with MNAH as a cofactor (Scheme 3). The name DT diaphorase comes from the previous nomenclature of NADH and NADPH as DPNH and TPNH, respectively, because this enzyme does not differentiate between the two cofactors. The same efficiency was observed with a nitroreductase enzyme (Knox et al. 1995). The authors speculated that the fact these enzymes do not distinguish between either NAD(P)H was the reason they could accept synthetic analogues.

Clark and coworkers have more recently tested the activity of cytochrome P450 BM3 and a mutant W1064S/R966D against BNAH and its *para*-methoxy derivative (Ryan et al. 2008). While the wild-type P450 showed no activity with the mimics for a hydroxylation reaction, the mutant gave rates of 23.3 and 14.7 nmol s^−1^ mg^−1^ of BM3 for BNAH and *p*-MeO-BNAH, respectively, compared to 30.4 and 34.5 nmol s^−1^ mg^−1^ of BM3 for NADPH and NADH.

**Scheme 4:**
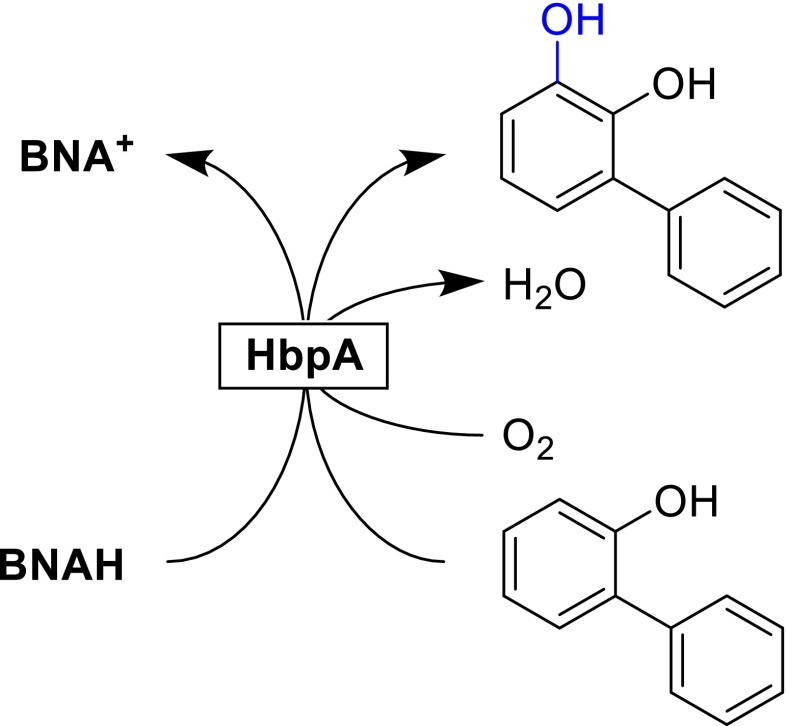
Hydroxylation catalyzed by HbpA using BNAH

The enzyme 2-hydroxybiphenyl 3-monooxygenase HbpA was also shown to use BNAH to form catechol derivatives (Scheme 4) (Lutz et al. 2004). Compared to the natural cofactor (NADH), the hydroxylation rate was reduced significantly whereas the oxidative uncoupling rate (i.e., the futile oxidation of the cofactor yielding H_2_O_2_ without phenol hydroxylation) was increased approximately tenfold.

**Scheme 5:**
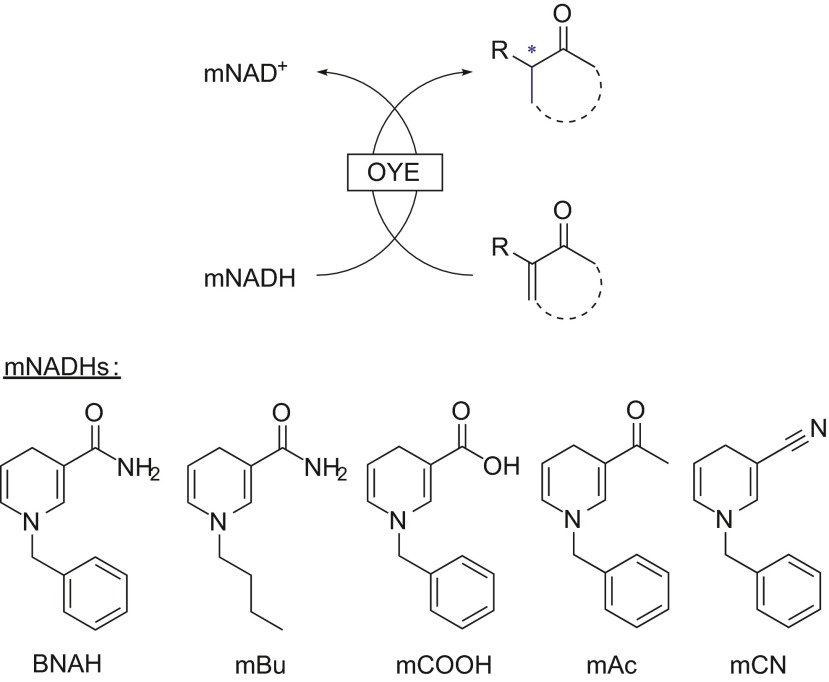
OYE-catalyzed asymmetric hydrogenation with synthetic biomimetics mNADHs

In a first study on ene reductases (ERs), three old yellow enzymes (OYEs) were screened against a panel of synthetic nicotinamide analogues varying the substituent X and group R from the structure in Fig. 1, shown in Scheme 5 (Paul et al. 2013).

Time course experiments showed different rates of reactions between each analogue and both natural cofactors. A more extensive screening of ERs with the same series of analogues was performed with detailed presteady state kinetic data to establish these differences (Knaus et al. 2016). The results showed that each ER gave a different rate of reaction depending on the cofactor analogue, and that in several cases, the biomimetics afforded higher *k*_cat_ values when compared to NADPH or NADH. While the *k*_cat_ values were higher for certain mimics with respect to the natural cofactors, the *K*_M_ values were also elevated, showing a lower affinity. Crystal structures obtained of XenA with the three analogues BNAH, mAc, mCOOH and natural cofactor NADPH showed the presence of a tryptophan residue that adopts an alternative conformation with the three analogues, thus reducing the volume in the active site (Knaus et al. 2016). This observation could partially explain the different rates. Another two studies on ERs with the BNAH analogue and derivatives showed similar results: certain biomimetics gave higher *k*_cat_ values with certain ERs and substrates with respect to NAD(P)H (Löw et al. 2016; Riedel et al. 2015).

**Scheme 6:**
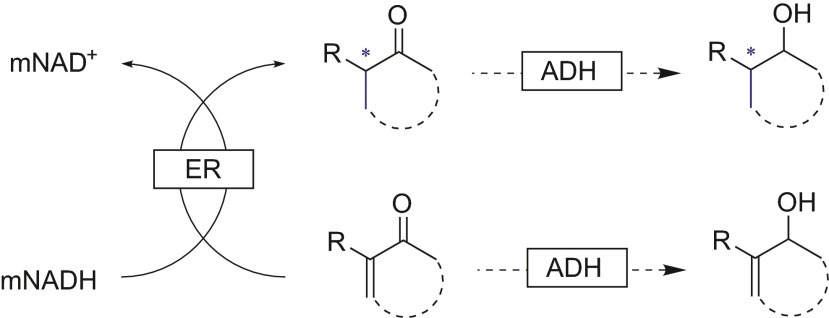
Ene-reductase-catalyzed asymmetric hydrogenation that could lead to an additional two side products due to the presence of ADHs, which are not active when using a biomimetic as the cofactor

The lack of ADH activity toward biomimetics previously mentioned can be turned into an advantage, for example when using crude cell extracts of OYE, where the presence of ADHs can lead to undesired side product with the over-reduction of the carbonyl group (Scheme 6) (Paul et al. 2013).

**Scheme 7:**
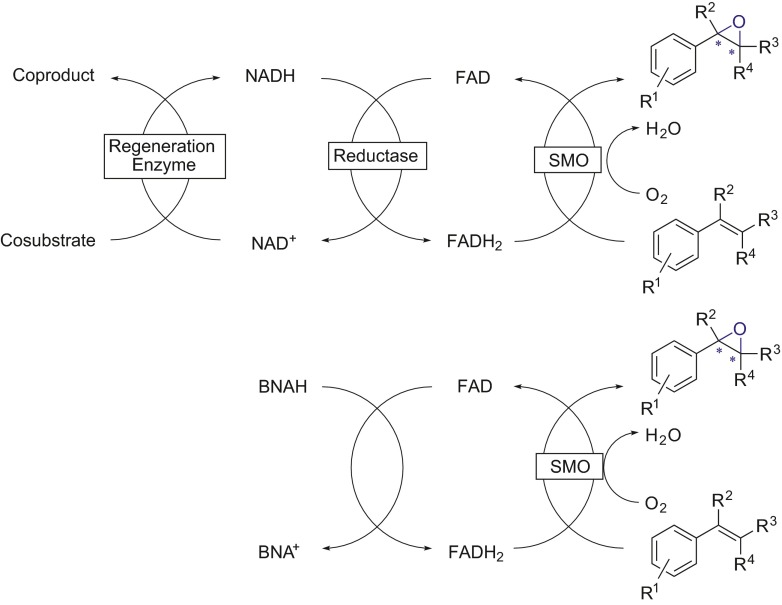
Simplified regeneration of SMOs (*bottom*) replacing the natural nicotinamide cofactor together with a corresponding enzymatic regeneration system (*top*) with BNAH

Synthetic nicotinamide analogues can also be used as a source of electrons to directly reduce the flavin cofactor in free solution. This usage was shown with a styrene monooxygenase (SMO) StyA, which usually requires a reductase, StyB, to reduce FAD from NADH in order to obtain reduced FADH_2_ (Paul et al. 2015). Using the biomimetic BNAH, a higher efficiency of the SMO-catalyzed process was demonstrated (Scheme 7) with electron transfer yields of up to 80 % for the asymmetric sulfoxidation of thioanisole derivatives.

**Scheme 8:**
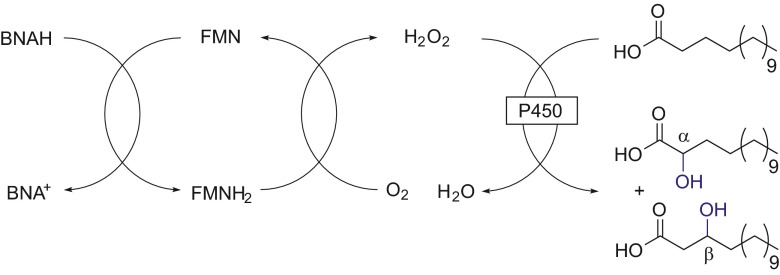
Production of hydrogen peroxide from the mimic-reduced FMN and reoxidation with molecular oxygen, allowing the P450-catalyzed hydroxylation of myristic acid

In a study with peroxygenases, BNAH was used to directly reduce FMN, which reoxidizes with molecular oxygen, producing hydrogen peroxide in the process to selectively hydroxylate the fatty acid myristic acid (Scheme 8) (Paul et al. 2014b).

## Recycling of biomimetics

The inactivity of dehydrogenases with synthetic nicotinamides has largely excluded their application in catalytic amounts. One alternative was explored early on by Fish and coworkers (Lo et al. 1999; Lo et al. 2001) using the organometallic catalyst [Cp*Rh(bpy)(H_2_O)]^2+^ for the formate-driven in situ regeneration of BNAH (and other mimics) from the corresponding oxidized forms. Although this catalyst system is efficient and highly selective, issues regarding the mutual inactivation of Cp*Rh-complexes and enzymes (Poizat et al. 2010) (Hildebrand and Lutz 2009) may severely limit the general applicability of this system. One promising solution could be the confinement of the transition metal complex, e.g., into a streptavidin, protecting both the complex and the biocatalysts from mutual inactivation (Kohler et al. 2013).

The group of Sieber has developed a regeneration system for BNA^+^ and MNA^+^ with the water-forming NADH oxidase from *Lactobacillus pentosus* (*Lp*Nox) (Nowak et al. 2015). This process represents the first enzymatic recycling system for oxidized synthetic analogues. The *k*_cat_ for MNAH and BNAH were reported to be 0.14 and 0.17 s^−1^, respectively compared to 43.4 s^−1^ for NADH, whereas the *K*_M_ are each 1.6 and 1.3 mM, compared to 17.9 μM for NADH. The recycling of reduced synthetic cofactors has until now only been performed with transition metals in situ such as the rhodium complex discussed above (Knaus et al. 2016), further enzymatic regeneration systems still remain unavailable as the classical glucose and formate dehydrogenases screened have not shown activity towards biomimetics.

## Conclusions

Synthetic nicotinamide cofactor analogues have been known for over 80 years; nevertheless, their journey into enzymatic processes is only beginning. Up to date, diaphorases, P450s, ERs, SMOs, and NADH oxidases have been demonstrated to accept these biomimetics. On the other hand, purified BVMOs, ADHs, GDHs, and FDHs have displayed no activity so far and other enzyme families remain to be investigated. Therefore, there is still a need for improved synthetic mimics as well as modified enzymes, such as dehydrogenases, that could be used in a regeneration system. The use of designed mimics used for certain targeted enzymes in a biorthogonal fashion can also be an advantage for selective syntheses using crude enzyme preparations.
